# Adaptive Graph Learning with Multimodal Fusion for Emotion Recognition in Conversation

**DOI:** 10.3390/biomimetics10070414

**Published:** 2025-06-25

**Authors:** Jian Liu, Jian Li, Jiawei Dong, Zifan Mo, Na Liu, Qingdu Li, Ye Yuan

**Affiliations:** 1 Institute of Machine Intelligence, University of Shanghai for Science and Technology, Shanghai 200093, China; liujian92@usst.edu.cn (J.L.); liuna_job@163.com (J.D.); liuna@usst.edu.cn (N.L.); liqd@usst.edu.cn (Q.L.); 2 Department of Information Technology, National Technical University Kharkiv Polytechnic Institute, 61000 Kharkiv, Ukraine; jian.li@cs.khpi.edu.ua; 3 School of Automation and Electronic Information, Xiangtan University, Xiangtan 411105, China; 202205801126@smail.xtu.edu.cn

**Keywords:** emotion recognition, graph neural networks, adaptive graph structure learning, conversational AI, transformer-based fusion

## Abstract

Robust emotion recognition is a prerequisite for natural, fluid human–computer interaction, yet conversational settings remain challenging because emotions are shaped simultaneously by global topic flow and local speaker-to-speaker dependencies. Here, we introduce GASMER—Graph-Adaptive Structure for Multimodal Emotion Recognition—a unified architecture that tackles both issues. It uses the correlation structure based on graph neural networks (GNNs) to model the complex dependencies in the conversation, while adaptively learning the graph structure for GNNs. The experiments indicate that our model has strong performance that outperforms all existing graph-based approaches, and remains competitive when compared to recent multimodal fusion models, underscoring the importance of combining fine-grained multimodal fusion with adaptive graph learning for conversational emotion recognition. On the IEMOCAP dataset, GASMER improves accuracy by 2.7% and the weighted F1-score by 3.6% compared to the best baseline. On the MOSEI dataset, it achieves a 1.2% gain in binary classification accuracy (ACC-2).

## 1. Introduction

**Figure 1:**
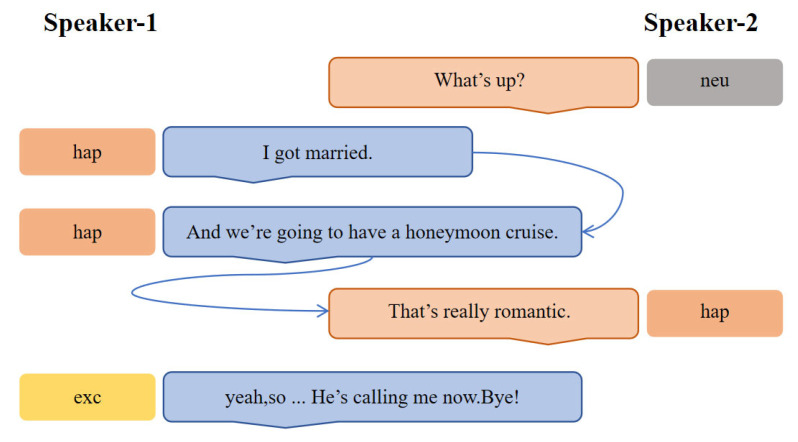
An example conversation that reveals the influence of the context and the utterance emotion of speaker-1 on the utterance of speaker-2.

Emotions expressed in conversation seldom emerge in isolation [1]; they are tightly coupled to both the thematic context and the affective cues of other speakers [2]. For example, during a discussion about upcoming holidays, the prevailing topic naturally primes participants toward joyful anticipation, making positive affect more likely. Conversely, in a heated debate, one speaker’s aggressive tone can quickly propagate frustration or defensiveness throughout the dialog [3]. Such cases highlight two key factors that any emotion-recognition model must capture [4]: contextual information (topic flow and discourse setting) and inter-speaker dependencies (how one utterance influences another) (as shown in Figure 1).

Treating a dialog as a graph naturally captures these dependencies, and graph neural networks (GNNs) have therefore become a popular choice for conversation modeling. Yet, practical obstacles remain. First, the graph structure is rarely given a priori; it must be inferred from the conversation itself. Second, naïvely constructing a fully connected graph presumes that every utterance influences every other one, incurring $O\left(n^{2}\right)$ edges and quickly becoming infeasible for long exchanges. Some studies sidestep this cost with a fixed-width context window, but a static neighborhood is seldom optimal because conversational influence is highly dynamic.

To overcome these limitations, we introduce GASMER—a GNN with Adaptive Structure for Multimodal Emotion Recognition. GASMER rests on two key ideas: Firstly, we introduce the modality fusion adapter (MFA), which can be embedded into each layer of the Transformer, enhancing the efficacy of visual and audio modalities to extract richer and more informative data. Secondly, we propose an adaptive graph structure learning approach that incorporates self-supervised tasks. By dynamically learning the graph structure based on the specific context, our method aims to provide a more efficient and effective solution for modeling conversations.

Recent advances in graph neural networks and multimodal learning have opened up new opportunities for modeling complex social signals in conversation. However, most existing approaches rely on fixed graph structures or treat modality fusion and structural modeling as separate stages. Our motivation in proposing GASMER is to unify these aspects through a dynamic and adaptive framework that is both context-aware and speaker-sensitive. This integrated perspective aligns with the growing interest in embodied AI and emotionally intelligent dialog systems.

In conclusion, we make the following contributions:

1. GASMER framework: We present a multimodal emotion-recognition model that simultaneously exploits contextual history and speaker-to-speaker dependencies through an adaptively learned graph;
2. Self-supervised graph module: We design a lightweight objective that infers dialog structure on-the-fly, eliminating the brittleness of preset graphs;
3. Layer-wise multimodal fusion: The proposed MFA integrates audio-visual signals inside the Transformer, yielding richer, more discriminative representations;
4. Outstanding results: GASMER achieves state-of-the-art performance among graph-based methods, and shows competitive results on the IEMOCAP and MOSEI benchmarks for multimodal emotion recognition.

## 2. Related Work

Building social chatbots and intelligent dialog systems that can engage in empathetic conversations has long been a central ambition of artificial intelligence [5]. Emotion recognition in conversation (ERC) is critical to this objective, and recent progress has been driven by the increasing availability of high-quality multimodal corpora such as CMU-MOSEI [6] and IEMOCAP [7].

### 2.1. Multimodal Emotion Recognition

Because of the high correlation observed between emotions and facial expressions [8], the methods using multimodal fusion to improve the effect of emotion recognition tasks are also being increasingly widely used [9]. Datcu et al. [10] integrate audio modality and visual modality for emotion recognition. Joshi et al. [11] adopt a graph neural network to model the inter/intra dependencies of speakers in conversation. Zadeh et al. [12] proposed the Tensor Fusion Network to model the intra- and inter-modality dynamics underlying multimodal sentiment analysis. Wollmer et al. [13] add contextual information to the multimodal task for emotion recognition. Sun et al. [14]; Li et al. [15] treat the conversation as a graph structure and model the contextual information via a graph neural network. Mao et al. [16] propose the concept of emotion dynamics to capture contextual information and use a multi-granular intertalk fusion approach across modalities to model the cross-modal emotion dynamics. Huang et al. [17] proposed to use Transformer to integrate visual and speech information at the model level. Liu et al. [18] proposed CapsGCN to represent the fusion of the multimodal model. Siriwardhana et al. [19] proposed a multimodal emotion recognition framework based on Transformer self-supervised feature fusion, using the trained self-supervised network to extract the features of multimodal information, including textual, audio, and visual features, and at the same time, using a method based on Transformer and the attention mechanism to capture the context of semantic connection between and within modalities. Tan et al. [20] utilized human facial images and EEG signals for multimodal recognition, used CNN to extract face features for classification and SVM-classified EEG signals, and finally obtained the final multimodal emotion classification results through multiple voting. Pandeya et al. [21] extracted face features using 3D-CNN and finally classified them via the late fusion method. Huang et al. [22] proposed the deep multimodal attention fusion method (DMAF), which used the difference and internal correlation between visual and semantic content to identify emotion through a hybrid fusion framework. Lian et al. [23] proposed a Transformer-based session-level multimodal emotion recognition framework containing two steps of context-independent discourse-level feature extraction and context-dependent multimodal feature extraction. One Transformer in the architecture is used to capture the temporal features of unimodal features, and the other cross-modal Transformer is used to learn the cross-modal interaction information on non-aligned multimodal features, and to perform multimodal feature fusion through the attention mechanism.

### 2.2. Graph Structure Learning

Zhao et al. [24] proposed GAUG-M, which models the weight of edges by calculating the inner product of the features of two nodes. Lim et al. [25] proposed AdaCAD, which considers both node features and graph structure while designing the transformation matrix. To enhance expression ability, Yuan et al. [26] used the cosine kernel to learn node feature representation and encode the local and global graph structure using the additional diffusion kernel. Gidaris et al. [27] created a KNN similarity graph using the cosine similarity of node features as a similarity measure. Wang et al. [28] extended Gidaris’ approach to create a new graph structure based on the cosine similarity of the node features in each layer of the GNN. Halcrow et al. [29] incorporated multiple similarity measures to create KNN graph structures. Zhang et al. [30] proposed to reduce the complexity by defining for each node the local neighborhood and assuming that these local neighborhoods are fully connected. Li et al. [31] generated a fully connected graph based on a bilinear similarity function with learnable parameters. Franceschi et al. [32] learned the Bernoulli distribution for each edge and used these distributions to create the graph structure. Yang et al. [33] updated the initial input structure according to the label and model predictions to increase homogeneity. Chen et al. [34] proposed an iterative approach that projects nodes onto the latent space and constructs an adjacency matrix with a latent representation. Qasim et al. [35] used an MLP as a projection layer to learn the graph structure. Kazi et al. [36] used a GNN as the projection layer and created graph structures separately using features from different layers. To solve the supervised hunger problem, Fatemi et al. [37] introduced a self-supervised task to guide graph generator learning.

Collectively, these studies underscore two trends crucial in our work: (i) the benefit of adaptive graph learning for capturing dynamic conversational structure and (ii) the effectiveness of fine-grained multimodal fusion for robust emotion recognition. GASMER builds upon and unifies these directions through a self-supervised, layer-wise multimodal GNN with an adaptively learned topology.

## 3. Proposed Method

In the ERC task, an important emotion recognition strategy involves modeling conversational scenarios. In a conversation involving multiple speakers, each speaker’s emotions are influenced by the context and the responses of other speakers. Inspired by this insight, we have primarily focused on two types of situational information to model the conversational scenario: contextual information and speaker-level dependency information.

In our model, we represent these two types of scenario information as follows: Global information—this aspect captures the impact of the overall context on the emotional state of the utterance. Local information—this aspect captures the influence of inter-speaker dependencies and intra-speaker dependencies on the emotional state of the utterance.

Global Information: Recognizing the importance of extracting contextual information and handling multimodal data, we advocate for the adoption of a unified model. In this study, we employ the Transformer encoder [38] to extract global information. To capture the full influence of context on each utterance, we depart from traditional encoding methods that involve adding positional encoding to input features, opting instead for a vanilla Transformer encoder. However, in preparation for the subsequent extraction of local information and the fusion of multimodal data, we introduce the modality fusion adapter (MFA) to be embedded within the Transformer encoder, enabling the learning of temporal information within the MFA.

Local Information: In binary or multi-party dialog systems, the information in adjacent utterances often exerts the greatest influence on emotions. We model the relationship between adjacent utterances through speaker-level dependent information, which is further categorized into inter-speaker and intra-speaker dependencies. Inter-speaker dependence refers to the emotional impact of one person on another in the conversation, while intra-speaker dependence represents the individual’s emotional influence within the conversation, also referred to as emotional inertia. Our approach shares similarities with DialogueGCN [39] and COGMEN [11]; we model the conversation as a graph, with each utterance representing a node and the relationships between nodes being represented by directed edges. We classify the edge relationships into two categories: the relationship between utterances spoken by the same speaker and the relationship between utterances spoken by different speakers. To learn the relationships between the nodes, we utilize the Relational Graph Convolutional Network [40] and Graph Transformer [41].

Additionally, to address challenges related to the learning difficulties caused by preset graph structure defects in graph neural networks, we have designed a self-supervised graph structure learning module to overcome this issue.

### 3.1. Overall Architecture

**Figure 2:**
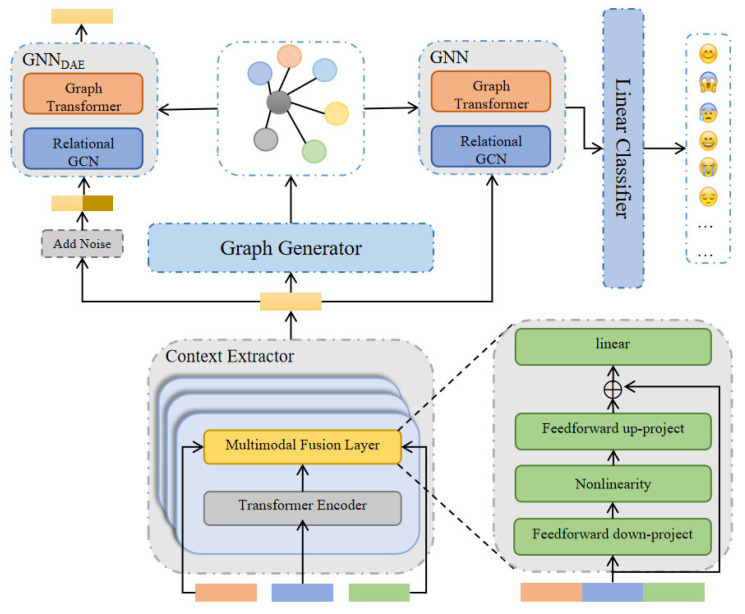
The overview of GASMER.

As illustrated in Figure 2, GASMER comprises the context extractor, graph generator, local feature extractor, and emotion classifier. Initially, the context feature extractor is employed to extract text, audio, and video features from the input utterance to acquire global context information. Diverging from prior work, we have integrated the modality fusion adapter into the Transformer encoder layer to combine text, audio, and video features, thereby enhancing the temporal information within the Transformer encoder. The features extracted by the context feature extractor serve as inputs for the graph generator to derive the graph structure, which, along with the graph node features, is fed into the graph neural network (GNN). The local feature extractor, leveraging GNN components like the Relational Graph Convolutional Network and Graph Transformer, captures inter-speaker and intra-speaker dependencies from the graph structure. Ultimately, the output features of the local feature extractor are utilized for emotion prediction through the emotion classifier, implemented as a linear layer.

### 3.2. Context Extractor

The context extractor inputs multimodal utterance features to represent each conversation utterance. The specific input form can be expressed as follows:

(1) $$U=\left[u_{1},u_{2},\ldots ,u_{i}\right]\;R^{n}$$

(2) $$u_{i}=u_{i}^{\left(a\right)},u_{i}^{\left(t\right)},u_{i}^{\left(v\right)}$$

where n is the number of utterances in a conversation, $u_{i}^{\left(a\right)}\;R^{d_{a}}$ is the audio feature of utterances, $u_{i}^{\left(t\right)}\;R^{d_{t}}$ is the text feature of utterances, and $u_{i}^{\left(v\right)}\;R^{d_{v}}$ is the video feature of utterances. Previous work has proven that the text modality is more indicative than other modalities, so we adopt the strategy of using the text modality as the input to the Transformer encoder layer and fusing other modalities in the modality fusion adapter. According to the vanilla Transformer encoding layer, we define a Query, a Key, and a Value vector to encode the input features in the following form:

(3) $$X=x_{t}=\left[u_{1}^{t},u_{2}^{t},\ldots ,u_{i}^{\left(t\right)}\right]$$

(4) $$Q_{i}=XW_{i,q}$$

(5) $$K_{i}=XW_{i,k}$$

(6) $$V_{i}=XW_{i,v}$$

where $W_{i,q},\;W_{i,k},\;W_{i,v}\;R^{d_{t}\times k}$. Scaled dot product attention captures the interaction relationship between Key and Query and outputs an attention coefficient graph $\alpha^{\left(h\right)}$ as follows:

(7) $$\alpha_{i}=\sigma_{j}\left(\frac{Q_{i}\left(K_{i}\right)}{\sqrt{k}}\right)$$

where $\alpha^{\left(h\right)}\;R^{n\times n}$ represents the attention coefficient of the individual attention head for each discourse in the following form:

(8) $$head_{i}=\alpha_{i}\left(V_{i}\right)\;R^{n\times k}$$

(9) $$H^{\prime }=\left[head_{1}⊕head_{2}⊕\ldots \;head_{h}\right]W$$

where $W\;R^{kh\times d_{t}}$, H represents the number of attention heads in the multi-head attention mechanism. The obtained $H^{\prime }\;R^{h\times d_{t}}$ performs the residual connection and enters the feedforward layer in the following form:

(10) $$H=LayerNorm\left(X+H^{\prime }\right)$$

(11) $$Z^{\prime }=ReLU\left(HW_{1}\right)W_{2}$$

(12) $$Z=LayerNorm\left(H+Z^{\prime }\right)$$

where $W_{1}\;R^{d_{t}\times m}$, $W_{2}\;R^{m\times d_{t}}$. The textual features provided by the Transformer encoder that contain the conversation context information are ${\left[z_{1},z_{2},\ldots ,z_{n}\right]}^{T}=Z\;R^{n\times d_{t}}$.

Different from the previous feature fusion method of simple concatenation, we embedded the modality fusion adapter into the Transformer encoder, fused text features, audio features, and video features, and learned the temporal information that is not included in the Transformer encoder. We refer to the traditional adapter [42] to connect the modality fusion adapter after each Transformer encoder layer.

We take the output text features Z of the Transformer encoder, the audio features of utterance, and the video features of utterance together as the input of the modality fusion adapter in the following form:

(13) $$x_{i}^{\left(avt\right)}=\left[u_{i}^{\left(a\right)}⊕z_{i}⊕u_{i}^{\left(v\right)}\right]\;\;R^{d}$$

(14) $$X=x^{\left(avt\right)}={\left[x_{1}^{\left(avt\right)},x_{2}^{\left(avt\right)},\ldots ,x_{n}^{\left(avt\right)}\right]}^{T}$$

where $d=d_{a}+d_{t}+d_{v}$. The obtained multimodal fusion feature X enters the modality fusion adapter for downward projection and upward projection to optimize the specific parameters of multimodal fusion, and finally, the feature dimension of X is mapped back to the dimension of text features. For the context extractor of layer j, the multimodal fusion is expressed as follows:

(15) $$X_{u}=\sigma \left(W_{u}X^{j-1}+b_{u}\right)$$

(16) $$X_{v}=W_{v}X_{u}+b_{v}$$

(17) $${X^{j}}^{\prime }=W\left(X_{v}+X^{j-1}\right)$$

(18) $$X^{j}=LayerNorm\left({X^{j}}^{\prime }\right)$$

where σ Sigmoid function, $W_{u},W_{v},W,b_{u},b_{v}$ are learnable parameters, and $X^{j-1}$ denotes the multimodal fusion adapter after  j−1 Transformer layers. At the same time, in order to better capture the temporal information, we follow the practice of AIM [43] and define the input of the multimodal fusion adapter as $X\;R^{b\times l\times d}$, where b is the size of the batchsize and l is the length of the conversation, so as to learn the temporal relationship between each utterance in the conversation. This explicit operation helps the model to enhance the temporal modeling and keep the number of parameters stable.

### 3.3. Graph Generator

By considering the significant influence of adjacent utterances on emotions during conversations and taking into account the typical conversation length characteristics, we have adopted a strategy to discretize the adjacency radius. This approach simplifies the conventional task of generating graph structures by focusing on classifying the adjacency radius of graph nodes. Through this simplification, we are able to generate adjacency vectors that effectively represent the graph structure. This method allows us to capture the emotional impact of adjacent utterances in conversations more accurately, leading to improved emotion modeling within dialog systems. We use the MLP as a graph-generator function:

(19) $$G_{MLP}\left(X;\theta_{G}\right)=Z$$

(20) $$P=softmax\left(Z\right)$$

(21) $$N=argmax\left(P\right)$$

where $X\;R^{n\times d_{t}}$ is the output of the context extractor, θ is the learnable parameters of MLP, $Z\;R^{n\times w}$ is the result vector of the graph node neighbor radius,  w is the number of categories after the discretization of the adjacent radius, and $N\;R^{n}$ is the last predicted node adjacency radius. Finally, the adjacency vector is generated through N, which considers the relationship between past N utterances and future N utterances.

### 3.4. Graph Neural Network Components

Considering the significance of speaker-level dependence information, we have leveraged graph-based models to represent these relationships. Within this framework, each utterance is associated with speaker-level and temporal dependencies, which are systematically interconnected through various relationship types. Specifically, if there are M speakers in a conversation, then there are $R=M\left(speaker\;of\;u_{i}\right)*M\left(speaker\;of\;u_{j}\right)*2\left(u_{i}\;occurs\;before\;or\;after\;u_{i}\right)=2M^{2}$ different relationships in the graph. This approach allows us to comprehensively capture the interplay between speaker-level dependence and temporal dynamics within conversations, enabling a more nuanced understanding of the underlying dependencies and facilitating the improved modeling of dialog interactions.

In our framework, we recognize that each speaker in a conversation is influenced by the utterances of other speakers. To model these dependencies effectively, we define clear relationships and employ the Relational Graph Convolution Network (R-GCN) to capture inter-speaker and intra-speaker dependencies, enabling the extraction of local information within the dialog context. The R-GCN operates based on the types of edges present in the graph structure, allowing it to leverage the information from adjacent nodes to update the representation of each node. This tailored approach enables us to capture the intricate relationships and dependencies between speakers in conversations, enhancing the overall understanding of speaker interactions and facilitating more accurate information extraction at the local level.

(22) $$x_{i}^{\prime }=W_{0}x_{i}+\sum_{r\;R}\sum_{j\;N_{r}\left(i\right)}\frac{1}{\left|N_{r}\left(i\right)\right|}W_{r}x_{j}$$

where $N_{r}\left(i\right)$ is the set of adjacent node indices of node i under the relation r R, $W_{0}$ and $W_{r}$ are learnable parameters, $\left|N_{r}\left(i\right)\right|$ are the normalized constants, and $x_{j}$ are the utterance level features from the context extractor.

To enhance the information extracted from the node features, we used GraphTransformer to process the node features obtained from the R-GCN in the following form:

(23) $$h_{i}^{\prime }={W_{1}x}_{i}^{\prime }+\sum_{j\;N_{r}\left(i\right)}\alpha_{i,j}W_{2}x_{j}^{\prime }$$

where $W_{1}$ and $W_{2}$ are learnable parameters and $\alpha_{i,j}$ is the attention coefficient calculated via scaled multi-head dot product attention:

(24) $$\alpha_{i,j}=softmax\left(\frac{{\left({W_{3}x}_{i}^{\prime }\right)}^{T}\left({W_{4}x}_{j}^{\prime }\right)}{\sqrt{d}}\right)$$

where $W_{3}$ and $W_{4}$ are learnable parameters and $\sqrt{d}$ is the scale coefficient.

For a concrete example illustrating how speaker-level relationships are represented in our constructed graph, please refer to the Appendix A.

### 3.5. Emotion Classifier

In the final stage of our framework, we employ an emotion classifier that utilizes the features extracted by the Graph Transformer. The obtained features are fed through a linear layer to predict the corresponding emotions associated with each node in the graph. The classification task loss is calculated using a SoftMax function to obtain the emotion probability distribution for each node. Subsequently, the cross-entropy loss is computed to measure the disparity between the predicted probability distribution and the actual emotions, enabling the model to learn and optimize its parameters for accurate emotion classification.

(25) $$h_{i}=ReLU\left(W_{1}{h_{i}}^{\prime }+b_{1}\right)$$

(26) $$P_{i}=softmax\left(W_{2}h_{i}+b_{2}\right)$$

(27) $$\hat{y_{i}}=argmax\left(P_{i}\right)$$

where $\hat{y_{i}}$ is the emotional label corresponding to the predicted utterance.

### 3.6. Self-Supervision Task

In the existing framework, it is feasible to establish a comprehensive pipeline for conversation emotion recognition utilizing the outlined scheme. However, relying solely on the loss of the classification task for supervising graph structure learning may not yield optimal results. Moreover, parameter fluctuations in the graph generator can significantly impact the accuracy of the overall conversation emotion recognition task. To mitigate these challenges, we introduce a self-supervised task and adopt a differentiated learning rate strategy to separate the training of the graph generator from that of other network structures.

To enhance the efficacy of graph structure learning, incorporating prior information into the graph generation task can be beneficial. The selection of appropriate prior information is crucial as it influences the inductive bias of the model learning process. In this context, we establish a prior graph structure based on the hypothesis: a graph structure suitable for predicting the characteristics of the node is also suitable for predicting the node label [37].

Firstly, considering an extreme case where the node features are the same as its node labels. In the scenario, the graph structure that is effective for predicting the features exhibits homogeneity. Due to the feature being equivalent to the label, the graph structure also demonstrates homogeneity in predicting the labels. As a result, the same graph structure that accurately predicts the features is inherently suitable for predicting the node labels as well.

In general, the node features may not be directly equivalent to the node labels. However, it is possible that a subset of the node features exhibits strong predictability for the labels. In such cases, the graph structure that is effective at predicting this subset of features is likely to be highly predictive for the labels as well. When a specific subset of features holds significant predictive power for the labels, the graph structure aligned with predicting these features also demonstrates a high level of homogeneity in relation to the labels. Consequently, this graph structure is also well-suited for predicting the node labels, due to the strong predictive relationship between the subset features and the labels.

Introducing prior information through a self-supervised task is a valuable approach for enhancing model learning. In this context, we employ a denoising autoencoder (DAE) as the self-supervised task. The denoising autoencoder shares the same structure as the GNN components utilized earlier in the model. It takes as input the noisy version of the node features, derived from the context feature extractor, and the adjacency vector generated by the graph generator. The autoencoder’s objective is to reconstruct the original, denoised node features by removing the added noise elements. We set idx as the corresponding index of the noise added elements and $X_{idx}$ as the index value, and set the loss function for the denoising task as follows:

(28) $$L_{DAE}=L\left(X_{idx},GNN_{DAE}{\left(\tilde{X},A;\theta_{GNN_{DAE}}\right)}_{idx}\right)$$

where A is the adjacency vector generated by the graph generator, L is the mean square error loss, $\tilde{X}$ is the node feature of the noisy version, and $\theta_{GNN_{DAE}}$ is a learnable parameter of the denoising autoencoder. Finally, our entire task loss function is as follows:

(29) $$L=L_{C}+\lambda L_{DAE}$$

where $L_{C}$ is the loss of emotion classification, $L_{DAE}$ is the denoising autoencoder loss, and λ is the importance coefficient.

## 4. Experiments

We compared the GASMER and baseline models on two widely used datasets to evaluate the effect of our proposed model, and performed sufficient ablation experiments to demonstrate the effectiveness of our proposed module.

### 4.1. Datasets

We selected two widely used datasets for emotion recognition: the Interactive Emotional Dyadic Motion Capture (IEMOCAP) dataset [7] and the Multimodal Opinion Sentiment and Emotion Intensity (MOSEI) dataset [6]. The detailed statistics of the two datasets are shown in Table 1.

MOSEI is a multimodal emotion recognition dataset using 22,860 movie clips from YouTube. This dataset is annotated with seven sentiment annotations (−3(highly negative) to +3(highly positive)) and six emotional labels—happiness, sadness, disgust, fear, surprise, and anger—for each sample. It is important to note that the emotional labels provided in different datasets are often different. IEMOCAP is a binary multimodal emotion recognition dataset, consisting of 7433 samples. We selected two versions of six emotion classifications (happiness, sadness, neutral, anger, excitement, and frustration) and four emotion classifications (anger, sadness, happiness, and neutral) for testing.

For CMU-MOSEI, in addition to the 7-class emotion classification task (ACC-7), we also conduct a binary classification task (ACC-2). In this setting, all samples with positive sentiment scores (greater than 0) are labeled as “positive”, and those with negative sentiment scores (less than 0) are labeled as “negative”. This binary setup is widely adopted in prior works to evaluate polarity-level emotion recognition.

### 4.2. Data Preprocessing

Like some previous works [44], for IEMOCAP, we used OpenSmile [45] to extract audio features, video features using the method of Baltrusaitis et al. [46], and text features using sBERT [47]. For MOSEI, we extracted audio features using the method of Delbrouck et al. [48] and used Librosa [49] to extract a specific number of filter sets. Video features were extracted using the method of Baltrusaitis et al. [46] and text features were extracted using sBERT.

### 4.3. Evaluating Indicator

For MOSEI, we use two evaluation settings: ACC-7 for 7-class emotion classification, and ACC-2 for the binary classification of sentiment polarity. The binary task distinguishes between positive and negative sentiments, as described in Section 4.1.

### 4.4. Baselines

We compared our method to baseline models in many emotion recognition tasks to comprehensively evaluate its performance. For the IEMOCAP, the specific evaluation results are shown in Table 2. The baseline models used include the Tensor Fusion Network (TFN) [12], MMGCN [50], Multiview Sequential Learning Memory Fusion Network (MFN) [51], DialogueRNN [52], DialogueGCN [39], Multimodal Dynamic Fusion Network (MM-DFN) [53], COGMEN [53], and Interactive Conversational Memory Network ICON [54]. For the MOSEI, the specific assessment results are shown in Table 3. The baseline models include the Tensor Fusion Network (TFN), COGMEN, Multimodal Factorization Model (MFM) [55], Low-rank Multimodal Fusion (LMF) [56], Interaction Canonical Correlation Network (ICCN) [57], Self-MM [58], and MMIM [59].

### 4.5. Experimental Settings

We use PyTorch (2.0.1) [60] as the training framework, PyG [61] to build the GNN components in our model, and Bayesian optimization for hyperparameter tuning. Our trained batch size is set to 64, the graph generator learning rate is set to 3 × 10^−5^, and the remaining components learning rate is set to 1 × 10^−4^. The audio feature size is 100, video feature size is 512, text feature size is 768, and fusion vector size is 768. In the loss function, we set the importance coefficient = 10.

### 4.6. Results

GASMER is significantly better than the SOTA among graph-based methods in all indicators. Compared to the previous SOTA among graph-based methods, GASMER increased the ACC-2 by 1.2% in MOSEI (vs. MMIM), ACC by 2.7% in IEMOCAP (vs. MM-DFN), and 3.6% for the weighted F1-score (vs. COGMEN). Specifically, Table 2 shows the comparison results on IEMOCAP(6-way), showing that GASMER significantly outperforms previous graph-based baseline models in the ACC and weighted F1-score. It is evident that the classification scores for happiness, sadness, and excitement emotions have significantly improved compared to the previous baseline model. This improvement can be attributed to the incorporation of the graph learning module in our approach. Unlike the previous baseline models, our model considers the impact of the graph structure, which contributes to the enhanced performance.

We acknowledge that more recent multimodal fusion approaches such as the work by Ryumina et al. [62] have achieved higher overall results on both IEMOCAP and MOSEI. However, their approach focuses primarily on gated attention mechanisms across modalities, while our method emphasizes the dynamic learning of speaker relationships and conversational structure via self-supervised adaptive graph construction. As such, GASMER continues to offer complementary benefits within graph-based modeling paradigms.

Table 3 presents the comparison results on MOSEI, indicating that GASMER outperforms the previous graph-based baseline models in terms of the ACC-2 and demonstrates comparable performance to the current SOTA in terms of the ACC-7. To better handle class imbalance, we report the Unweighted Average Recall (UAR) or IEMOCAP(6-way), which computes the average recall across all classes, giving equal weight to each class regardless of its sample size. The results show that GASMER consistently achieves high UAR scores, demonstrating its robustness across uneven class distributions. Overall, GASMER consistently outperforms prior graph-based models in most instances, highlighting its superiority in emotion recognition tasks and validating the effectiveness of the GASMER framework.

### 4.7. Analysis

**Figure 3:**
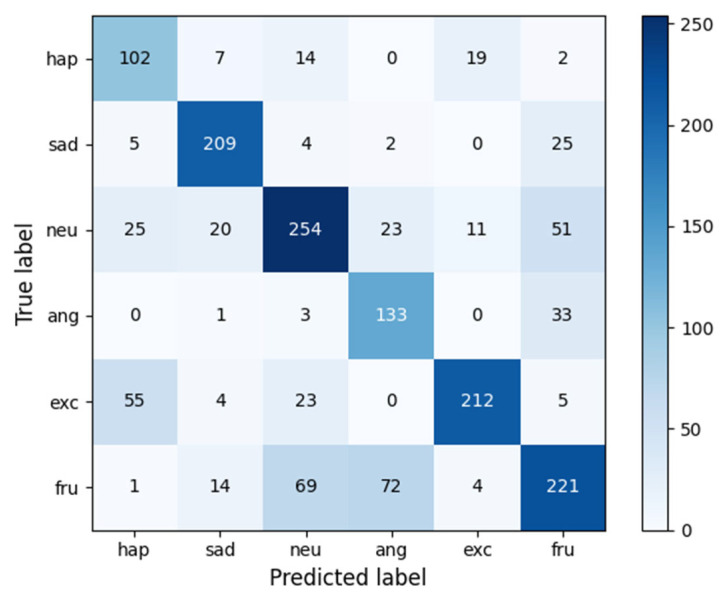
Confusion matrix for IEMOCAP(6-way).

Upon analyzing the predictions made by our model, we observed a considerable improvement in classifying similar emotions compared to previous baseline models. However, there are still certain emotions, such as anger and frustration, that our model struggles to classify accurately. Additionally, due to the substantial proportion of neutral samples, several instances are wrongly classified as neutral. To illustrate this, we have included the model prediction results for IEMOCAP(6-way) in Figure 3 and calculate the recall as shown in Table 4.

**Figure 4:**
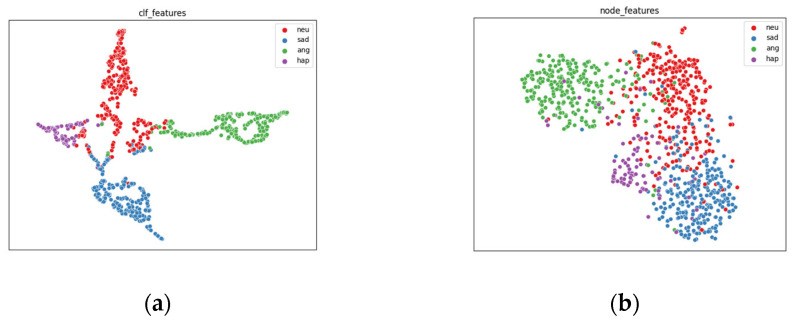
Take IEMOCAP(4-way) as an example, UMAP visual comparison, (**a**) is the feature before the GNN component, (**b**) is the feature after the graph learning component. It can be seen that the characteristics after the graph learning component form a better emotion cluster, showing the importance of GNN component and graph learning module.

To confirm the impact of GASMER’s graph learning module on the node feature representation, we utilized UMAP [63] to visualize the multimodal fusion representation output by the context extractor and the feature representation after the graph learning module. Figure 4 clearly demonstrates that the features following the graph learning module exhibit improved emotion clusters, highlighting the significance of capturing conversational local dependence through the graph learning module for emotion recognition.

### 4.8. Ablation Study

We conducted a series of ablation experiments on GASMER, and the specific results are presented in Table 5. In our ablation study, we treat the text modality (T) as the base configuration. This is because prior research and our own experiments show that text features typically carry the most semantically discriminative information for emotion recognition tasks. As such, we do not perform ablation on audio-only (A), video-only (V), or audio-plus-video (A + V) configurations without text, since our full model architecture is built upon the text backbone. Instead, we evaluate the impact of adding audio (A) and/or video (V) on top of text to examine multimodal complementarity. By systematically removing one or several modalities, we aimed to assess the impact of multimodal information on model performance. Our findings indicate that the exclusion of modality information leads to a decline in model performance, underscoring the necessity of leveraging multimodal information for effective emotion recognition tasks. Moreover, the results demonstrate the complementary nature of audio, text, and video modality information.

Interestingly, we observed that audio modality information plays a more significant role than video modality information, with instances where the addition of video modality information even negatively affects the results. This could be attributed to noise in the video modality and its lack of alignment with other modalities. However, GASMER consistently achieves notable performance when utilizing all modalities, showcasing its capability to capture complex interrelationships between modalities.

Furthermore, upon testing the removal of the MFA layer and graph learning (GL) module from GASMER, we observed a decrease in performance, highlighting the effectiveness of these components in multimodal emotion recognition tasks. While we primarily describe the experimental results on IEMOCAP due to space constraints, similar trends were also observed on MOSEI.

## 5. Discussion

In this study, we proposed GASMER, a graph-adaptive multimodal emotion recognition framework that integrates audio, video, and text modalities through self-supervised graph structure learning. Our experiments on IEMOCAP and MOSEI demonstrate that GASMER achieves superior performance compared to existing methods. Ablation experiments confirm the complementary role of multimodal inputs and the effectiveness of the proposed modality fusion adapter (MFA) and adaptive graph learning (GL) modules. Further visualization using UMAP highlights the improved emotion clustering enabled by the graph structure, demonstrating its contribution to contextual understanding.

We also performed a detailed error analysis. The model shows notable improvement in classifying emotions such as happiness and excitement, but still struggles with confusion between similar emotions like anger and frustration. Additionally, the overrepresentation of neutral samples contributes to prediction bias. These observations emphasize the importance of balanced datasets and more discriminative modeling for fine-grained emotions.

Despite these advancements, the current system operates in an offline mode and relies heavily on global contextual information. For real-world deployment, developing real-time emotion recognition capabilities remains a key challenge. Moreover, the reliance on accurate modality alignment and the performance sensitivity to video noise are areas for future enhancement.

## 6. Conclusions

We presented GASMER, a novel graph-adaptive multimodal emotion recognition framework designed for conversational settings. The model unifies multimodal feature fusion and speaker-level dependency modeling through a self-supervised graph learning mechanism. GASMER demonstrates competitive or superior performance on benchmark datasets, supported by extensive experiments and analyses.

In future work, we plan to (i) improve the model’s ability to distinguish subtle emotional variations through fine-grained modeling and (ii) develop methods to support real-time contextual emotion recognition for interactive and intelligent systems.

## Figures and Tables

**Table 1:** Detailed segmentation information for IEMOCAP and MOSEI.

Dataset	Train	Valid	Test	All
IEMOCAP	5146	664	1623	7433
MOSEI	16,327	1871	4662	22,860

**Table 2:** Results on IEMOCAP(6-way). WF1: Weighted F1-score, computed with class support as weights.

Model	IEMOCAP							
	Happy	Sad	Neutral	Angry	Excited	Frustrated	Avg	
	F1 ↑	F1 ↑	F1 ↑	F1 ↑	F1 ↑	F1 ↑	ACC ↑	WF1 ↑
TFN	33.7	68.6	55.1	64.2	62.4	61.2	58.8	58.5
MMGCN	42.3	78.6	61.7	69.0	74.3	62.3	-	66.2
MFN	34.1	70.5	52.1	66.8	62.1	62.5	60.1	59.9
DialogueRNN	32.8	78.0	59.1	63.3	73.6	59.4	63.3	62.8
DialogueGCN	42.7	87.5	63.5	64.1	63.1	**66.9**	65.2	64.2
ICON	32.8	74.4	60.6	68.2	68.4	66.2	64.0	63.5
COGMEN	51.9	81.7	**68.6**	66.0	75.3	58.2	68.2	67.6
MM-DFN	42.2	78.9	66.4	**69.7**	75.5	66.3	68.2	68.1
GASMER	**64.2**	**85.4**	67.9	66.5	**80.3**	62.7	70.9	71.2

**Table 3:** Results on MOSEI.

	MOSEI							
	TFN	COGMEN	MFM	LMF	ICCN	Self-MM	MMIM	GASMER
ACC-7 ↑	50.2	43.9	51.3	48.0	51.6	-	54.2	**54.3**
ACC-2 ↑	82.5	84.3	84.4	82.0	84.2	85.1	85.9	**87.1**

**Table 4:** Recall and UAR or IEMOCAP(6-way).

Recall						UAR
Happy	Sad	Neutral	Angry	Excited	Frustrated	
70.83%	85.31%	66.15%	78.24%	70.90%	58.01%	71.57%

**Table 5:** Ablation experiments on the IEMOCAP dataset, all evaluation indicators use F1-score (%). The results illustrate the effectiveness of the MFA layer and graph learning module.

	Modalities	T	A + T	T + V	A + T + V
IEMOCAP 6-way	Actual	65.6	68.4	64.7	71.2
	*w*/*o* MFA	63.6	64.7	63.2	64.8
	*w*/*o* GL	63.9	67.2	64.9	68.2
IEMOCAP 4-way	Actual	81.4	83.2	79.9	85.2
	*w*/*o* MFA	80.0	81.3	81.2	83.4
	*w*/*o* GL	80.7	83.3	80.6	83.9

## Data Availability

Data and materials are available here: https://github.com/liujianwgx/Adaptive-Graph-Learning-with-Multimodal-Fusion-for-Emotion-Recognition-in-Conversation-Code (accessed on 4 June 2025).

## Appendix A

To better demonstrate how we model a conversation into a graph and represent speaker-level relationships in the graph, we provide an example to describe the relationship between utterances in a conversation. In this example, we assume that there are two speakers, speaker1 and speaker2, who engage in a conversation consisting of five utterances. The adjacency radius N for each utterance is set to 2. $u_{i-2}$, $u_{i}$, and $u_{i+2}$ represent the utterances of speaker1, while $u_{i-1}$ and $u_{i+1}$ represent the utterances of speaker2. After modeling this conversation into a graph with relationships, its specific form is presented in Table A1.

**Table A1:** The relationships between each node are represented by (→) for future utterances and (←) for past utterances.

Node	Intra-Speaker Relationship	Inter-Speaker Relationship
$u_{i-2}$	$u_{i-2}\leftarrow u_{i-2}$ $,\;u_{i-2}\to u_{i}$ $,\;u_{i-2}\to u_{i+2}$	$u_{i-2}\to u_{i-1}$ $,\;u_{i-2}\to u_{i+1}$
$u_{i-1}$	$u_{i-1}\leftarrow u_{i-1}$ $,\;u_{i-1}\to u_{i+1}$	$u_{i-1}\leftarrow u_{i-2}$ $,\;u_{i-1}\to u_{i}$ $,\;u_{i-1}\to u_{i+2}$
$u_{i}$	$u_{i}\leftarrow u_{i-2}$ $,\;u_{i}\leftarrow u_{i}$ $,\;u_{i}\to u_{i+2}$	$u_{i-2}\leftarrow u_{i-1}$ $,\;u_{i-2}\to u_{i+1}$
$u_{i+1}$	$u_{i+1}\leftarrow u_{i-1}$ $,\;u_{i+1}\leftarrow u_{i+1}$	$u_{i+1}\leftarrow u_{i-2}$ $,\;u_{i+1}\leftarrow u_{i}$ $,\;u_{i+1}\to u_{i+2}$
$u_{i+2}$	$u_{i+2}\leftarrow u_{i-2}$ $,\;u_{i+2}\leftarrow u_{i}$ $,\;u_{i+2}\leftarrow u_{i+2}$	$u_{i+2}\leftarrow u_{i-1}$ $,\;u_{i+2}\leftarrow u_{i+1}$

Table A2 shows the types of relationships existing in this conversation.

**Table A2:** The types of relationships between the two speakers in the conversation.

Relationship Type	Temporal Relationship	Relationship
1	Past	$u^{\left(speaker1\right)}\leftarrow u^{\left(speaker1\right)}$
2	Past	$u^{\left(speaker1\right)}\leftarrow u^{\left(speaker2\right)}$
3	Past	$u^{\left(speaker2\right)}\leftarrow u^{\left(speaker1\right)}$
4	Past	$u^{\left(speaker2\right)}\leftarrow u^{\left(speaker2\right)}$
5	Future	$u^{\left(speaker1\right)}\to u^{\left(speaker1\right)}$
6	Future	$u^{\left(speaker1\right)}\to u^{\left(speaker2\right)}$
7	Future	$u^{\left(speaker2\right)}\to u^{\left(speaker1\right)}$
8	Future	$u^{\left(speaker2\right)}\to u^{\left(speaker2\right)}$
